# Biodistribution of adeno‐associated virus type 2 carrying multi‐characteristic opsin in dogs following intravitreal injection

**DOI:** 10.1111/jcmm.16823

**Published:** 2021-08-21

**Authors:** Kissaou T. Tchedre, Subrata Batabyal, Melissa Galicia, Darryl Narcisse, Sourajit Mitra Mustafi, Ananta Ayyagari, Sai Chavala, Samarendra K. Mohanty

**Affiliations:** ^1^ Nanoscope Technologies LLC Arlington Texas USA; ^2^ Nanoscope Therapeutics Inc Bedford Texas USA

**Keywords:** biodistribution, MCO, qPCR, rAAV, retinal gene therapy

## Abstract

Gene therapy of retinal diseases using recombinant adeno‐associated virus (rAAV) vector‐based delivery has shown clinical success, and clinical trials based on rAAV‐based optogenetic therapies are currently in progress. Recently, we have developed multi‐characteristic opsin (MCO), which has been shown to effectively re‐photosensitize photoreceptor‐degenerated retina in mice leading to vision restoration at ambient light environment. Here, we report the biodistribution of the rAAV2 carried MCO (vMCO‐I) in live samples and post‐mortem organs following intraocular delivery in wild‐type dogs. Immunohistochemistry showed that the intravitreal injection of vMCO‐I resulted in gene transduction in the inner nuclear layer (INL) but did not induce detectable inflammatory or immune reaction in the dog retina. Vector DNA analysis of live body wastes and body fluids such as saliva and nasal secretions using quantitative polymerase chain reaction (qPCR) showed no correlative increase of vector copy in nasal secretions or saliva, minimal increase of vector copy in urine in the low‐dose group 13 weeks after injection and in the faeces of the high‐dose group at 3–13 weeks after injection suggesting clearance of the virus vector via urine and faeces. Further analysis of vector DNA extracted from faeces using PCR showed no transgene after 3 weeks post‐injection. Intravitreal injection of vMCO‐I resulted in few sporadic off‐target presences of the vector in the mesenteric lymph node, liver, spleen and testis. This study showed that intravitreal rAAV2‐based delivery of MCO‐I for retinal gene therapy is safe.

## 1 INTRODUCTION

Retinitis pigmentosa (RP) refers to disorders characterized by degeneration of photoreceptors in the eye, which hinders visual ability by absence of photoactivated electrical signals in retina, and non‐transmission of those signals to the visual cortex^1^, ^2^, ^3^, ^4^ The prevalence of RP is ~80,000 patients in the US, out of which 20,000 patients have vision 20/200 or less. RP is most often inherited as an autosomal recessive trait with large number of cases having this form of inheritance.^1^, ^5^ Further, vision loss increases with ageing,^6^ which is a major concern since our population is living longer. Current clinical treatments are primarily focussed on slowing down disease progression,^7^ since there is not a FDA‐approved therapy that can halt the degeneration.^8^ Importantly, other than retinal prostheses, there are no clinically meaningful therapies that can restore vision once vision loss has ensued.^9^

Retinal implants are available for late‐stage RP patients under a Humanitarian Device Exemption (Argus II, Second Sight) and are indicated for use in one eye for patients with severe to profound RP, defined as bare light or no light perception in both eyes. However, only partial restoration of vision is possible due to inherent limitations to the retinal implant device. Also, a complex, invasive surgical procedure is involved, can only be used in one eye, requires rigorous training with behaviourial experts and includes a number of warnings and precautions related to electromagnetic interference. The ability of optogenetics to address the unmet need in RP has received considerable attention^10^ for its potential clinical utility, and unlike retinal implants, invasive surgery is not required. Optogenetics can be delivered to both eyes and offers the theoretical advantage of better resolution and one‐time administration. Also, while direct electrical stimulation approaches require mechanical contact of electrodes to the retinal cells, indirect stimulation approaches such as optogenetic stimulation^11^ do not necessitate such physical contact.

Classic gene replacement therapy halts retinal degeneration by replacing a defective gene with a functioning copy. This approach works best when there is minimal photoreceptor degeneration to preserve these cells and is limited in advanced cases where few photoreceptors remain. Currently, there are more than 60 gene mutations are associated with RP. Thus, a classic gene replacement therapy approach would require a multitude of gene replacement therapies to benefit all RP patients. Unlike classical gene replacement therapy, optogenetic therapy is gene mutation agnostic and does not require viable photoreceptor cells. Recently, we have developed an optogenetic therapy, multi‐characteristic opsin (MCO), which effectively re‐photosensitizes photoreceptor‐degenerated retina in mice leading to vision restoration at ambient light environment.^12^ Compared to other opsins, MCO has the advantage of functioning in ambient light and broad visible spectrum.^12^ Significant photocurrent is generated in MCO‐sensitized cells at white light intensity levels close to ambient light conditions without compromising the fast kinetics required to form vision. Owing to ambient light sensitivity, no external device‐based light stimulation is needed for MCO‐activation, thus eliminating potential phototoxicity. Further, MCO is polychromatic opsin that has broad activation spectrum, and therefore, subjects with MCO‐sensitized retina will have potential to regain vision at different colour environments.

The development of recombinant adeno‐associated viral (rAAV) vectors provides excellent vehicles for efficacious delivery and long‐term expression of gene therapy molecules, thus opening up new vistas for curing degenerative diseases. Several in vitro and in vivo systems are used for preclinical studies to produce information assuring safe administration of the investigational drug to humans.^13^ The MCO‐encoding gene, with distal CMV promoter and ON‐bipolar cell‐specific mGluR6‐enhancer along with mCherry as a reporter and enhancer, is packaged in an adeno‐associated virus type 2 (AAV2) vector for transducing retinal cells. Intravitreal injection of the rAAV carried MCO (vMCO‐I) transduces retinal cells and is preferentially expressed in retinal ON‐bipolar cells. Retinal ON‐bipolar cells are now activated and depolarized in response to light. The newly photo‐sensitized retinal cells have shown to drive retinal circuitry functions and visually guided behaviour.

The relevance of the vMCO‐I in vision restoration and study outcomes are fundamental for successful translation of optogenetics‐based technologies in human.^13^ Determining the AAV vector biodistribution is often conducted prior to early‐phase clinical trials for the safety.^13^, ^14^, ^15^ To determine whether the distribution of the vector may pose a risk to the patient, the level of genomes should be quantified in major organs as well as other tissues or fluids pertinent to the disease, gene therapy vector, transgene and route of administration.^15^ Though biodistribution studies in mice at different time points after intravitreal injection of different doses of vMCO‐I have been conducted, a study in a larger animal model that is more anatomically and physiologically relevant is required to justify first‐in‐human dosing. This will minimize uncertainty surrounding the scaling of vector dose from animal to human. The large size of the canine eye, the volume of the vitreous and its consistency and the anatomy of the retina that comprises a cone rich fovea‐like central region in the canine macula is close to that of the human. Further, disease metrics are well established for the human and canine diseases, and for both, the same methods of assessment are used. In addition, surgical interventions and doses of viral vectors in the dog can be easily translated to the human. Here, we report the biodistribution of the vMCO‐I in live samples, targeted retina and non‐targeted organs following intraocular delivery in wild‐type dogs. Our results indicate that, after the intraocular delivery of the vMCO‐I, off‐target presence of vMCO‐I is limited. AAV2‐based delivery of MCO to retina and evaluation of dose‐dependent MCO expression in targeted tissue (ie retina) is key to establish a safer therapeutic dose.

## 2 MATERIALS AND METHODS

### 2.1 Vector construct of ambient light activatable multi‐characteristic opsin

Multi‐characteristic opsin (MCO) gene under metabotropic glutamate receptor mGluR6 with reporter mCherry (mGluR6‐MCO‐I‐mCherry) was designed, constructed using rDNA technology and cloned at the restriction sites (BamH I and SalI) of pAAV‐MCS vector (Figure S1). The cloned mGluR6‐MCO‐I‐mCherry sequence was validated by sequencing and sequence alignment. After validating the cloned sequence, the plasmid construct was used for production of vMCO‐I.

### 2.2 Preparation of animals and intravitreal injection conditions

All animals were cared for and treated in accordance with the Nanoscope Technologies sponsored CRO's IACUC approved protocol (# NS‐1702). The monocular intravitreal injections, similar to those that would be used in human eyes, were performed in three different groups of wild‐type beagle dogs. Each group consisted of four dogs (two males and two females). Intravitreal injections of the right eyes were performed with the control vehicle (75 µl, 8.6 × 10^12^ Viral Genomes/ml, ie VG/ml) administered to Group 1 dogs, the high‐dose (75 µl, 8.6 × 10^12^ VG/ml) vMCO‐I administered to Group 2 dogs and the low‐dose (75 µl, 1 × 10^12^ VG/ml) vMCO‐I article administered to the Group 3 dogs (Table S1A). Stagger 1 dogs were injected on 1 day, and then, the stagger 2 dogs were injected on a following day. The viral titre used in the intravitreal injection was determined by qPCR with standard curve generated by linearized plasmid DNA. Each dog was sedated with atropine (0.04 mg/kg), acepromazine (0.04 mg/kg) and butorphanol (0.1 mg/kg) administered by either intramuscular or subcutaneous injection. Topical neomycin‐polymyxin B‐dexamethasone drops and 1% tropicamide drops were instilled three times at 5‐min intervals. Anaesthesia was induced with propofol (2 mg/kg) by intravenous injection. The dogs were intubated and maintained on isoflurane. Intravitreal injections were performed with the dogs in dorsal recumbency with the head positioned such that the right eye faced upward. Following trimming of the upper eyelid lashes, the globe surface and periocular regions were prepped with baby shampoo diluted with saline (1:5), followed by two preparations with povidone‐iodine solution diluted with saline (1:50). Topical 0.5% proparacaine was instilled prior to performing the intravitreal injections. A sterile surgical towel was placed along the lower eyelid and maxillary region, and then, a wire lid speculum was placed to maintain the eyelids in an open position. The superior temporal, perilimbal conjunctiva was grasped with Manhattan Eye and Ear forceps to stabilize the globe. Hamilton syringe with a 27‐gauge hypodermic needle was preloaded with the 75 µl of AAV vehicle control (AAV2 without the MCO transgene) or 75 µl of two different (high and low) doses of vMCO‐I solution. Intravitreal injections were performed, 6–8 mm behind the limbus at the superior temporal aspect of the sclera. The injections were administered into the mid‐vitreous. A subconjunctival injection of 0.15 ml of triamcinolone (40 mg/ml) was administered to the injected eye, and two drops of topical neomycin‐polymyxin B‐dexamethasone ophthalmic suspension were applied. The dogs were recovered from anaesthesia and extubated.

### 2.3 Tissue extraction

At the pre‐scheduled time point (13 weeks post‐injection), the dogs were euthanized and different organs (heart, liver, spleen, kidney, mesenteric, mandibular, testis/ovary) from each dog in the three different groups were collected. The organ tissues were kept in the 1.8 ml cryovials and stored at −80°C. Each vial was properly labelled with study number, animal identification number, date of extraction and name of organ. Faeces, saliva and nasal secretions were collected at 1, 3, 13 weeks post‐injection (Table S1B).

### 2.4 Immunofluorescence microscopy

Dog tissue samples were fixed in 10% neutral‐buffered formalin and embedded in paraffin wax. De‐paraffinized tissue sections (6 mm thick) were used for immunohistochemistry staining. The immunohistochemistry reagents included a washing solution (0.5% Triton in 1× PBS), a blocking solution (4% goat serum in washing solution), primary antibodies solutions against mCherry, PKC‐alpha, interferon‐gamma and CD45 (Table S7), and secondary antibodies such as Dylight 488, Alexa Fluor 488 and Alexa Fluor 568 (Table S8). Table S9 shows the dilution of the respective antibodies. Tissue sections of both control and vMCO‐I‐injected dogs were initially reacted with primary antibody (Tables S7 and S9). Tissue sections were washed three times and treated with secondary antibodies (Tables S8 and S9) to detect the expression of the transgene or the induction of an immune reaction. The staining was performed as described by Christopher Kerfoot et al.^16^ Fluorescence imaging was carried out on the immunoassayed slices using 20X oil objective under Olympus confocal microscope (Fluoview FV1000).

### 2.5 DNA extraction

Genomic DNA was extracted from tissue samples using the Phenol/chloroform DNA extraction technique.^17^ DNA from faeces, saliva, nasal secretion and urine was extracted using the Thermo Fisher Scientific GeneJET Genomic DNA Purification Kit (cat# K0722) according to the manufacturer's protocol.

### 2.6 Quantitative PCR analysis

qtuantitative polymerase chain reaction was performed using Takara AAVpro^™^ Titration kit standard (cat# 6233) and Fisher Scientific Company's Master Mix, qPCR (Applied Biosystems) and Power Up SYBR Green Master Mix (cat# A25776). 50X Primer Mix was prepared as follows: AAV Forward ITR Primer 5 µl, AAV Reverse ITR primer 5 µl, and water 15 µl. The qPCR reaction mix consisted of SYBR green Premix 12.5 µl, 50X Primer Mix 0.5 µl, water 7 µl, and template DNA (~10 ng) 5 µl. Primers for the pAAV2 plasmid ITR and MCO were as follow: The AAV2 ITR qPCR is based on the forward primer (forward ITR primer, 5′‐GGAACCCCTAGTGATGGAGTT‐3′) and the reverse primer (reverse ITR primer, 5′‐CGGCCTCAGTGAGCGA‐3′). Real‐time PCR was performed on the Applied Biosystems QuantStudio 3 real‐time PCR System (Applied Biosystems) using assays specific for ITR. qPCR conditions were as shown in the Figure S2. Samples were analysed in duplicate for vector copy number/ng DNA by the absolute quantification method using standard curves. Preparation of the standard curve was performed following the manufacturer's reference guide.

### 2.7 Statistical analysis

Data are expressed as the mean of vector copy number (Av.) ± standard deviation (SD). One‐way ANOVA analysis was carried out. Further, for comparisons of number of vector copies between baseline and different time points, the outcome measures for each group were analysed by a two‐tailed Student's *t*‐test with Bonferroni correction using Statistical Package for the Social Sciences (SPSS) version 24. A *p*‐value of <0.05 was deemed significant. For inter‐group comparisons, the outcome measures (vector copy/ng DNA) were analysed using the same method. However, to test inter‐group differences, the regression analyses used generalized linear models with baseline values as covariate.

## 3 RESULTS

The main objective of this study was to determine the biodistribution of the vMCO‐I in live samples, targeted retina and non‐targeted organs after intravitreal injections in wild‐type dogs. The schematic of the vector used (Figure S1) shows that it contains two AAV2 inverted terminal repeats (ITR) that flank the cloning sites. The study comprised of three groups of dogs with each group consisting of four dogs (two males and two females in each group) at the euthanization time point of 13 weeks (Table S1A,B). The primary end points in this study were the immunohistochemistry examination, transgene expression in the retina and vector genome detection in live body wastes (urine, faeces) and body fluids (saliva and nasal secretions) as well as in non‐targeted tissues (lung, liver, kidney, mandibular/mesenteric lymph nodes, heart, spleen and testis/ovary).

### 3.1 Immunohistochemistry and transgene expression

**FIGURE 1:**
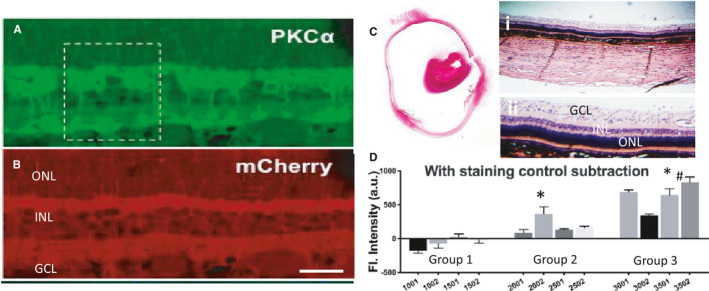
Immunohistochemical analysis of MCO‐I‐mCherry expression in retina of vMCO‐I‐injected dog eye. Immunostained images of (A) Bipolar cell marker, PKCα; (B) MCO‐mCherry expression. (C) H&E‐stained eye section showing the whole retina along with eye ball (Left); (i) retina and (ii) zoomed retina showing the layers (Right). (D) Quantification of mCherry fluorescence intensity measured from five different areas in the inner nuclear layer (INL) for individual dog retina in each dose group. Av ± SD. Group 1: 8.6 × 10^12^ VG/ml AAV2‐Vehicle; Group 2: 8.6 × 10^12^ VG/ml vMCO‐I; Group 3: 1.0 × 10^12^ VG/ml vMCO‐I. *p < 0.05 between Group 1 and others; ^#^ *p* < 0.05 between Group 2 and Group 3. *N* = 4 dogs/Group and five regions in retina/dog

For quantifying transgene expression (MCO‐I) in targeted retinal cells (Bipolar cells), we assessed for reporter (mCherry) fluorescence in the paraffin‐embedded sections of vMCO‐I (or AAV vehicle)‐injected dog retina using immunohistochemistry. Figure 1A shows image of retina immunostained with PKCα (Bipolar cell marker). The mCherry expression in inner nuclear layer (INL) of the retina of vMCO‐I‐injected dog eye is evident in Figure 1B. Haematoxylin and eosin‐stained eye section of the whole retina along with the eye ball is shown in Figure 1C. In Figure 1D, we show quantification of mCherry fluorescence intensity measured from five different areas in the INL for individual dog retina in each dose group. The fluorescence intensity in staining control (with secondary antibody, but without primary antibody) was subtracted from the measured fluorescence intensity values of each group. Although the AAV vehicle control (Group 1: 8.6 × 10^12^ VG/ml AAV2‐Vehicle) did not exhibit any reporter characteristic fluorescence, the vMCO‐I‐treated groups (Groups2 and 3) showed statistically significant increase in transgene expression in INL. Inexplicably, the high‐dose group (Group 2: 8.6 × 10^12^ VG/ml vMCO‐I) showed lower expression than the low‐dose group (Group 3: 1.0 × 10^12^ VG/ml vMCO‐I).

**FIGURE 2:**
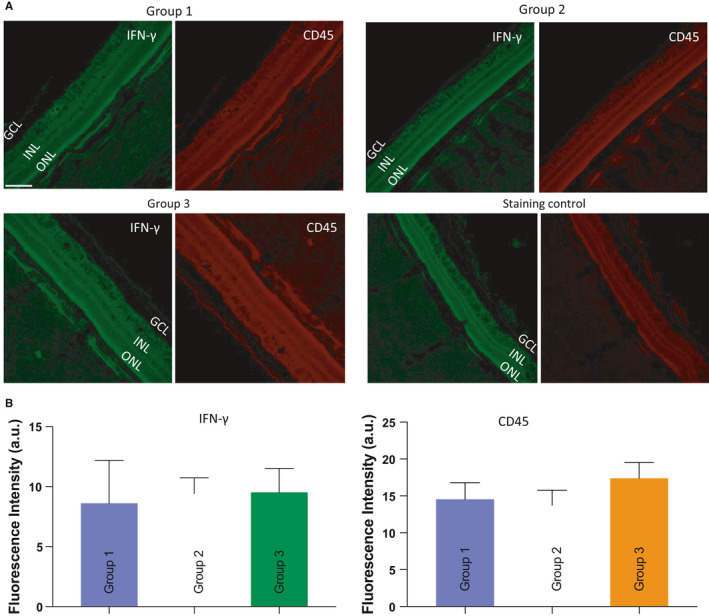
Immunohistochemical analysis of retina tissue response to intravitreal injection of AAV2. (A) Representative immunostained images of Interferon‐gamma and CD45 in different Groups: (Group 1) 8.6 × 10^12^ VG/ml AAV2‐Vehicle; (Group 2) 8.6 × 10^12^ VG/ml vMCO‐I; (Group 3) 1.0 × 10^12^ VG/ml vMCO‐I. Scale bar: 100 µm. GCL: ganglion cell layer; INL: inner nuclear layer; ONL: outer nuclear layer. (B) Quantitative analysis of immunostained images of interferon‐gamma and CD45 in different groups. Average ± SD. *N* = 4 dogs/Group and five regions in retina/dog

One major hurdle in using viral vectors for in vivo gene therapy is the development of host cellular immune responses to the vector, which may lead to the elimination of transgene expression.^18^ In order to determine immune response subsequent to intravitreal injection of vMCO‐I, we carried out immunostaining of retina with inflammatory cytokine (Interferon‐γ) and immune cell marker (CD45). Figure 2A shows IFN‐γ and CD45 immunostained images of retina of dogs of different treatment groups as well as the staining control. Absence of dose‐correlated or significant increase in IFN‐γ (green) signal or cellular CD45 (red) signal suggests that the intravitreal vMCO‐I injection does not lead to inflammatory cytokine or immune cells during MCO‐I transduction (Figure 2B).

### 3.2 Biodistribution of vMCO‐I in live samples

**FIGURE 3:**
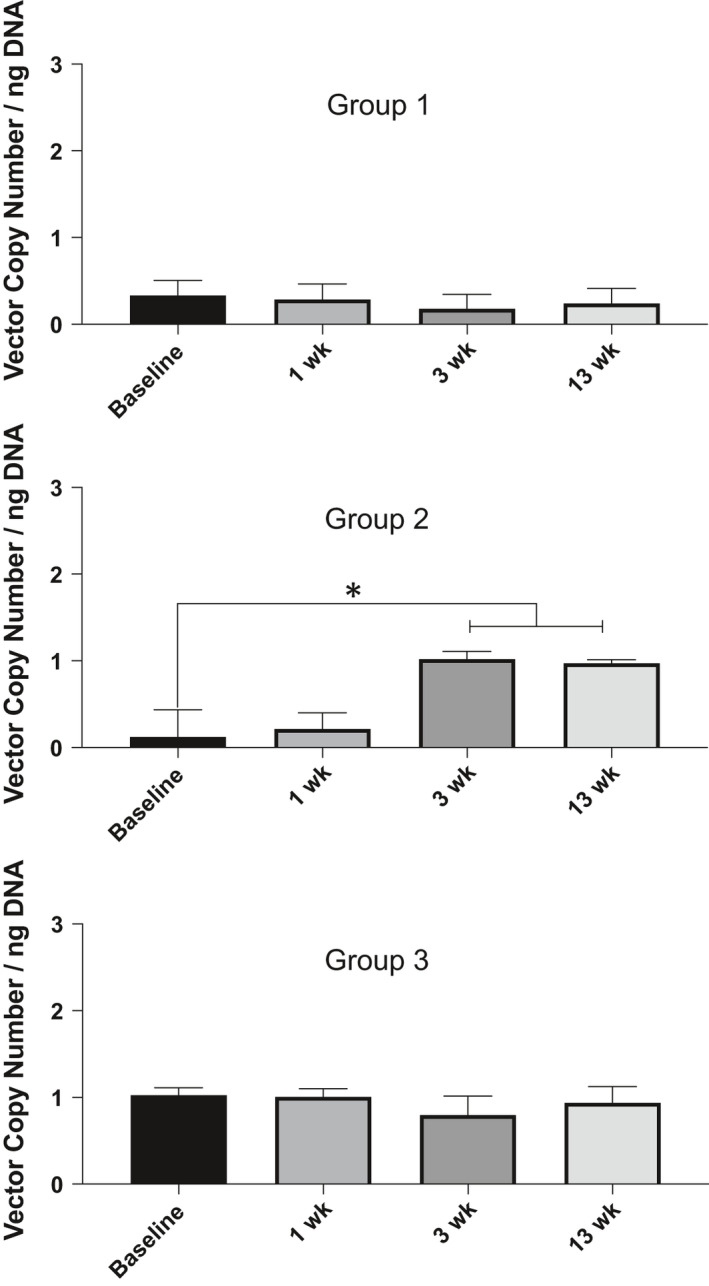
Longitudinal quantification of AAV2 in faeces of dogs before and after intraocular injection. qPCR detection of vector sequences in dogs' faeces. Summary of qPCR detection of vector copy number in: Group 1, Group 2, Group 3 dog faeces. Group 1: 8.6 × 10^12^ VG/ml AAV2‐Vehicle; Group 2: 8.6 × 10^12^ VG/ml vMCO‐I; Group 3: 1.0 × 10^12^ VG/ml vMCO‐I. Data are expressed as the mean of vector copy number, Av. ± SD. *p < 0.05. However, the average values are within error range of qPCR assay, which is attributed to sensitivity and variation in sample handling

**FIGURE 4:**
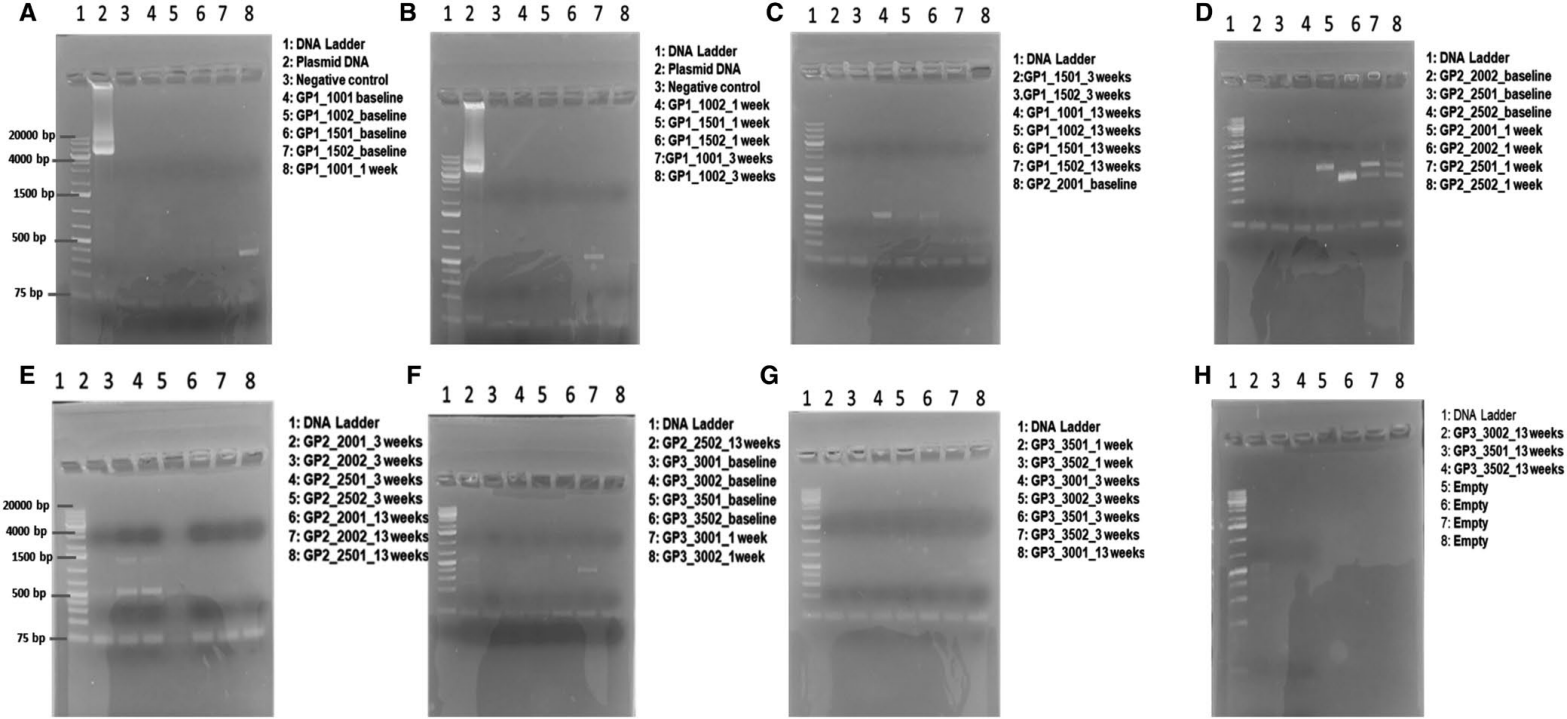
Polymerase chain reaction (PCR) Analysis of AAV2 in dog faeces. PCR detection of vector sequences in faeces. DNA samples were extracted from the faeces of dogs (Groups 1, 2, & 3). Group 1 (GP1): 8.6 × 10^12^ VG/ml AAV2‐Vehicle; Group 2 (GP2): 8.6 × 10^12^ VG/ml vMCO‐I; Group 3 (GP3): 1.0 × 10^12^ VG/ml vMCO‐I. The ITR segment of the vector gene was amplified for references. Representative results of the agarose gel electrophoresis of the PCR products were presented Gels (A‐H). No product was detected around 4 Kb as compared to the positive control (lane 2 in Gels A & B)

In order to understand the biodistribution of vMCO‐I and its clearance, we assayed for its presence in body wastes (urine and faeces) and body fluids (saliva and nasal secretions). Table S2 shows the longitudinal measurements of vector copy in dogs' faeces at baseline and three different time points (1, 3, 13 week) after intravitreal injection. For these measurements, DNA samples were extracted from faeces of dogs, wherein the ITR segment of the vector gene was amplified. Though ‘+’ implies vector amplification in qPCR, the average values are within error range of qPCR assay, which is attributed to sensitivity and variation in sample handling. Analysis of Groups 1, 2, 3 faeces samples showed no significant pre‐ to post‐injection variation over time for AAV vehicle‐injected or low‐dose vMCO‐I‐injected group (Figure 3). However, in high‐dose vMCO‐I‐injected dogs, qPCR detection of vector sequences shows increased vector copy in faeces at 3 and 13 weeks after injection, albeit at very low level (~1 copy/ng DNA). Tables [Link], [Link], [Link] show SPSS‐based analysis of differences in presence of vector DNA in faeces at different time points within Groups 1, 2 and 3, respectively. The progressive increase in vector copy/ng of DNA between 0 (baseline) and after injection time points can be seen only in Group 2 (high‐dose vMCO‐I, Table S11). Generalized linear model (Value = M1*Time point + Constant) was used for these analyses. SPSS‐based analysis was also conducted to determine difference between groups at different time points based on generalized linear model with Baseline as covariate (ie Value = M1*Group + M2*Baseline + constant). The significance values show higher values of Vector copy/ng of DNA in faeces at 13^th^ week after injection in the Groups 2 and 3 as compared to Group 1 (Table S13). To further confirm if there is any vector copy in faeces, PCR was performed on DNA samples from faeces. Representative images of the agarose gel electrophoresis of the PCR products are presented in Figure 4A‐H. No product (virus) was detected in the faeces samples around 4 Kb as compared to the positive control (lane 2 in panels A & B of Figure 4).

**FIGURE 5:**
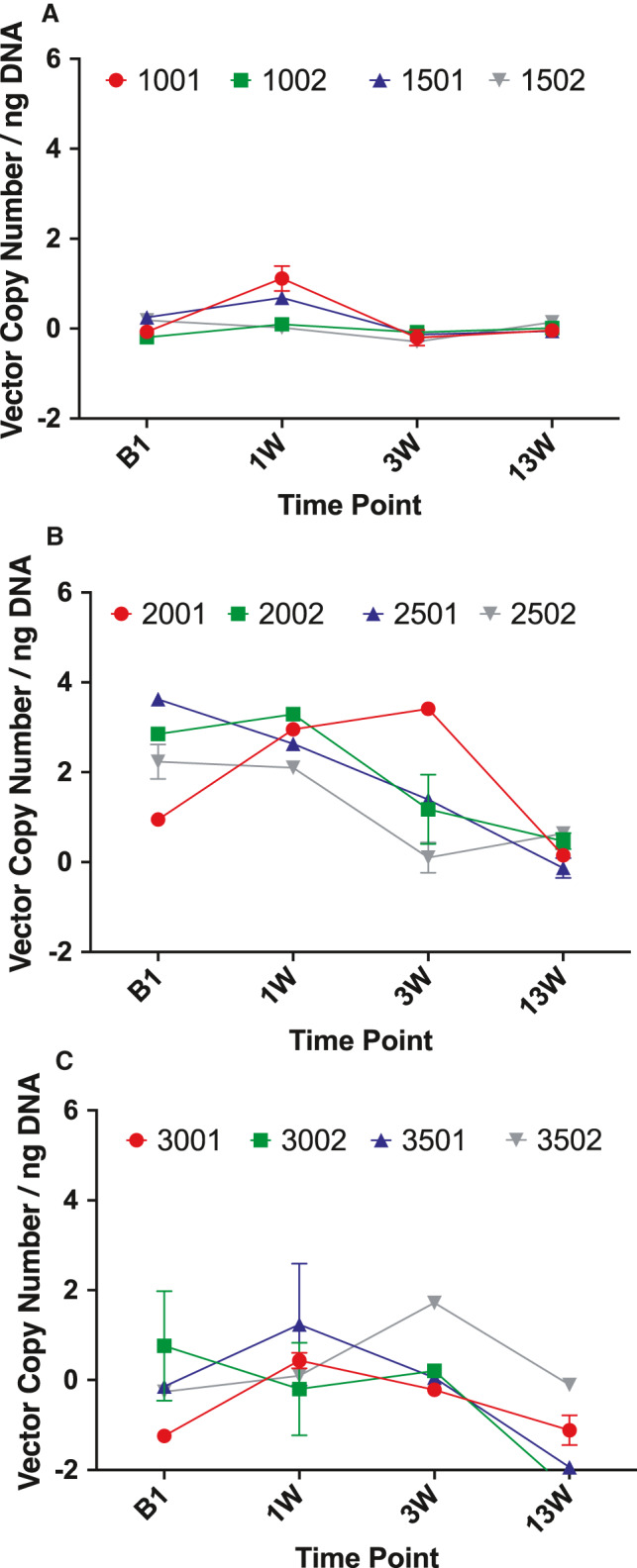
Quantitative Comparison of AAV2 in saliva. qPCR detection of vector sequences in dogs' saliva. Summary of qPCR detection of vector copy number in Group 1, Group 2, Group 3 dog saliva. (A) Group 1: 8.6 × 10^12^ VG/ml AAV2‐Vehicle; (B) Group 2: 8.6 × 10^12^ VG/ml vMCO‐I; and (C) Group 3: 1.0 × 10^12^ VG/ml vMCO‐I. Data are expressed as the mean of vector copy number, Av. ± SD. The average values are within error range of qPCR assay, which is attributed to sensitivity and variation in sample handling

The saliva samples of all groups showed non‐significant increase of qPCR signal of the vMCO‐I ITR from pre‐ (baseline) to post‐injection (week 1, 3, 13) as shown in Table S3. The observed ‘+’ amplification of ITR can be attributed to natural, exposure of the animals to AAVs. Though ‘+’ implies vector amplification in qPCR, the average values are within error range of the qPCR assay, which is attributed to sensitivity and variation in sample handling. In Figure 5, we show the qPCR‐based quantification of vector copy number in Groups 1–3 in dogs' saliva during baseline and post‐injection period. In Group 2 (high‐dose vMCO‐I) saliva samples, there was rather a decrease in measured vector copy number (vMCO‐I) from baseline to post‐injection time points. Additionally, the nasal secretions from any group of animals did not show presence of vMCO‐I. In Table S4, we show results of longitudinal measurements of vector sequence (via amplification of ITR segment) in nasal secretion of dogs injected with AAV vehicle or vMCO‐I. No amplification of ITR segment of the vector gene was observed in any of the groups or at any investigated time points (Table S4).

**FIGURE 6:**
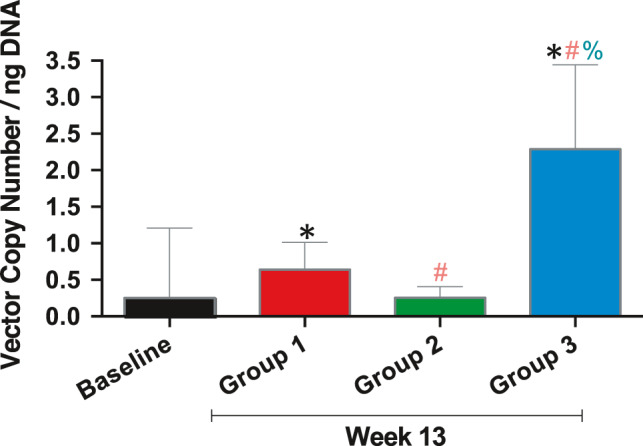
Quantitative polymerase chain reaction analysis of AAV2 in Urine of dogs. Summary of qPCR detection of vector copy number in urine from Groups 1, 2 &3 dogs. Group 1: 8.6 × 10^12^ VG/ml AAV2‐Vehicle; Group 2: 8.6 × 10^12^ VG/ml vMCO‐I; Group 3: 1.0 × 10^12^ VG/ml vMCO‐I. Data are expressed as the mean of vector copy number (Av.) ± SD. *p < 0.05 between baseline and groups; ^#^ *p* < 0.05 between Group 1 and other groups; and ^%^ *p* < 0.05 between Group 2 and 3

Detection of vector DNA in dog urines 13 weeks after intravitreal injection in Groups 1, 2 and 3 was carried out. Table S5 shows positive (+ve) vector amplification after intravitreal injection in all three groups. In Figure 6, we show qPCR analysis of vector copy number in urine of dogs intravitreally injected with AAV2 vehicle (Group 1) or vMCO‐I (low or high dose). The detected vector copy number in low‐dose vMCO‐I‐injected group (Group 3) was found to be higher than that injected with high dose (Group 2). Further, the vector copy number in Group 2 was lower than the AAV vehicle‐injected control group (Group 1).

### 3.3 Biodistribution of intravitreally injected AAV2 in non‐targeted tissues

**FIGURE 7:**
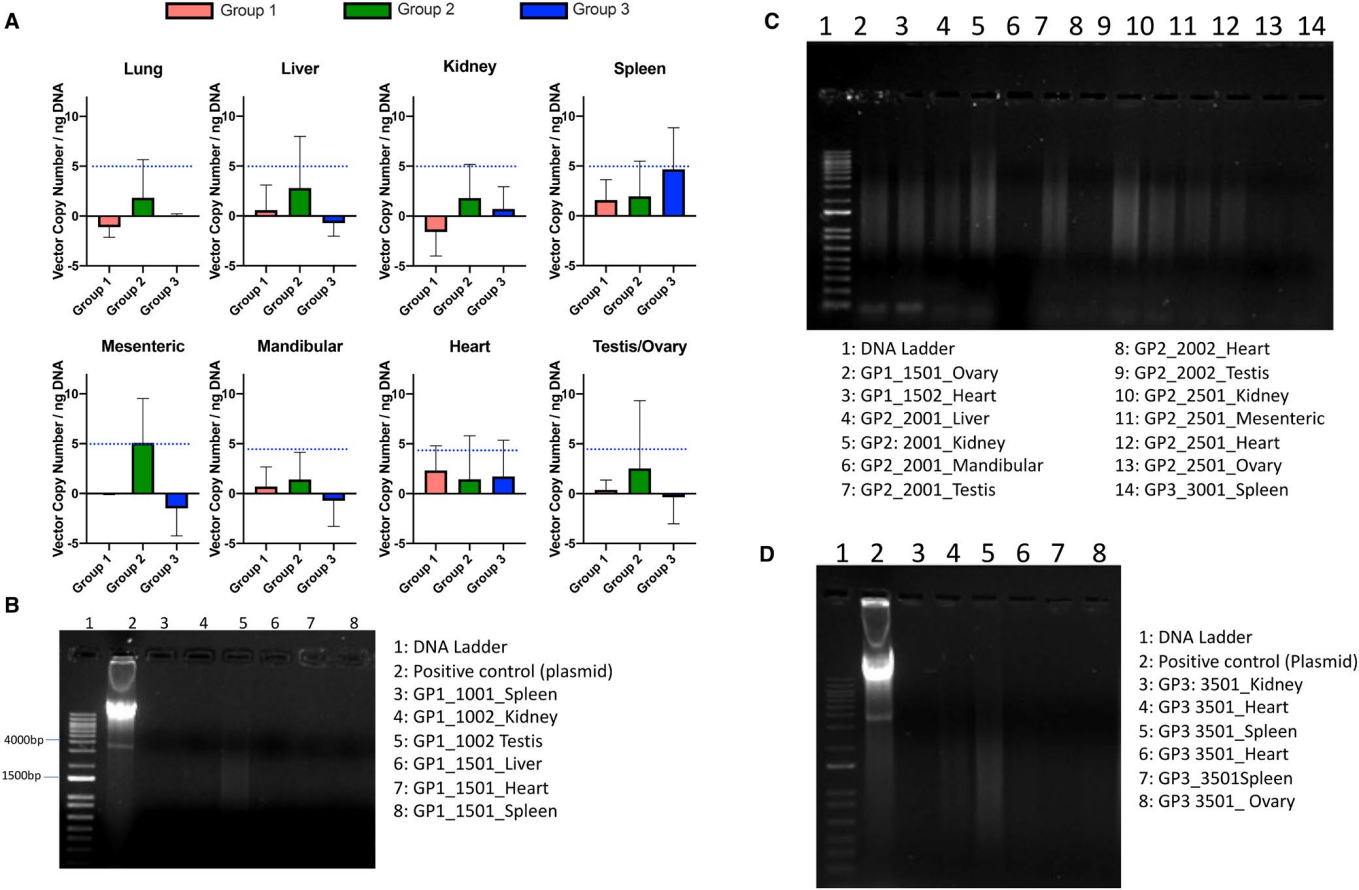
Biodistribution of intravitreally injected AAV2 in non‐targeted organs. (A) Summary of qPCR detection of vector copy number in dogs' tissues: Group 1: 8.6 × 10^12^ VG/ml AAV2‐Vehicle; Group 2: 8.6 × 10^12^ VG/ml vMCO‐I; Group 3: 1.0 × 10^12^ VG/ml vMCO‐I. Data are expressed as the mean of vector copy number. Av. ± SD. Except for mesenteric tissue, one‐way ANOVA analysis is not significant between the groups. However, the average values are within error range of qPCR assay, which is attributed to sensitivity and variation in sample handling. Limit of detection (LOD = 5 vector copy) is shown by dotted horizontal lines. (B‐D) PCR detection of vector sequences in various tissues after necropsy of dogs. Group 1 (GP1): 8.6 × 10^12^ VG/ml AAV2‐Vehicle; Group 2 (GP2): 8.6 × 10^12^ VG/ml vMCO‐I; Group 3 (GP3): 1.0 × 10^12^ VG/ml vMCO‐I. The ITR segment of the vector gene was amplified for references. Representative results of the agarose gel electrophoresis of the PCR products. No product was detected around 4 Kb as compared to the positive control

We assayed for the biodistribution of vMCO‐I using genomic DNA from non‐targeted tissues of distant organs (Lung, Liver, Kidney, Spleen, Mesenteric/Mandibular lymph node, Heart, and Testis/Ovary). No gross abnormalities were evident in any of the organs tissues. In negative control Group 1, qPCR results did not show any significant presence of vector genomes. Some of the signals were detected on the liver, heart, spleen and ovary of dog‐1501 and in the heart and mandibular lymph node in dog‐1502 (Table S6). However, as shown in Figure 7A, the vector copy / ng DNA was <2.2 in these tissues. To determine if +ve amplification implies presence of vector DNA in qPCR, or the average values are within error range of qPCR assay (due to sensitivity and variation in sample handling), PCR was performed (Figure 7B‐D). No apparent band corresponding to the expected product (~4 kD) was observed (Figure 7B‐D) implying absence of the vector.

For the dogs in Group 2 (high‐dose vMCO‐I), the qPCR signals were detected in the liver, kidney, mandibular lymph node and testis of dog‐2001 (Table S6). In dog 2002, the mesenteric lymph node, heart and testis showed signals of the presence of the virus ITR. In dog‐2501, lung, kidney, mesenteric lymph node, heart, spleen and ovary showed some positive results. No virus ITR signals were detected in any of the dog‐2502 organs (Table S6). In Group 3 (low‐dose vMCO‐I), in male dogs, the presence of virus ITR was detected in the spleen, and testis; however, in the female group, the virus signal was detected additionally in heart apart from spleen and ovary as per the qPCR analysis (Table S6). Since there was no consistence of +ve amplification in any organ in all the animals of any group, average with standard deviation was plotted and subjected to one‐way ANOVA test. As shown in Figure 7A, except mesenteric lymph node, no significant differences were observed between the groups. Finally, to confirm presence of the vector, PCR was performed on all tissue samples that showed positive results from the qPCR analysis. The plasmid construct (pAAV‐MCS‐mGLUR6‐MCO‐I‐mCherry) containing ITR was used as positive control. None of these samples exhibit any presence of expected ITR band (Figure 7B‐D). Further, no vector genomes were detectable in the gonads or ovaries of these dogs from PCR analysis, indicating that the risk of germ‐line transmission of vector DNA is very low, in accordance with two previous studies.^19^, ^20^

## 4 DISCUSSION

Recombinant adeno‐associated virus 2 (rAAV2) vectors have been used extensively as efficient gene delivery vehicles for the treatment of retinal diseases.^21^, ^22^, ^23^ Currently, there are over hundred AAV isolates and more than a dozen of them have been cloned for development as gene therapy vectors.^24^, ^25^, ^26^ Among these, serotype 2 (AAV2) has been the most extensively examined and has natural tropism towards retinal cells,^27^ skeletal muscles,^28^ neurons,^29^ vascular smooth muscle cells^28^, ^30^ and hepatocytes.^31^ AAV2 entry into the cell is mediated by three cell receptors: heparan sulphate proteoglycan (HSPG), aVβ5 integrin and fibroblast growth factor receptor 1 (FGFR‐1).^32^, ^33^, ^34^ HSPG functions as a primary receptor, whereas aVβ5 integrin and FGFR‐1 have a co‐receptor activity and enable AAV to enter the cell by receptor‐mediated endocytosis.^35^, ^36^ However, the function of these receptors is still not fully understood.^37^, ^38^ Earlier reports showed that HSPG functions as the primary receptor, though its abundance in the extracellular matrix can scavenge AAV particles and impair the infection efficiency.^37^, ^38^ Although AAV2 is the most popular serotype in various AAV‐based research, it has been shown that other serotypes can be more effective as gene delivery vectors. For instance, AAV6 appears much better in infecting airway epithelial cell,^39^, ^40^ AAV7 presents very high transduction rate of murine skeletal muscle cells (similarly to AAV1 and AAV5), AAV8 is superb in transducing hepatocytes,^41^, ^42^, ^43^ whereas AAV1 and 5 were shown to be very efficient in gene delivery to vascular endothelial cells.^44^, ^45^ In the brain, most AAV serotypes show neuronal tropism, whereas AAV5 also transduces astrocytes.^46^, ^47^ Very recently, it was reported that rAAV8 efficiently transduces murine photoreceptors.^43^, ^48^ Over the past 20 years, adeno‐associated virus (AAV) has emerged as a promising delivering vector for viral gene therapy.^49^, ^50^, ^51^ AAV is a parvovirus, which is a family of small, non‐enveloped viruses containing a single‐stranded linear DNA genome of about 5 kb; the wild‐type virus is replication‐deficient, requiring a helper virus in order to reproduce.^52^ In humans, AAVs have not been found to be pathogenic. This fact, along with the tendency for the genomes of recombinant AAV vectors to remain as episomal concatemers, rather than integrating into the host genome (reducing the risk for insertional mutagenesis), makes AAV a relatively safe gene therapy vector for testing in the clinic.^53^

To date, there are very few published studies of biodistribution following intravitreal injection of AAV2.^14^, ^54^, ^55^, ^56^ A time course that spans the peak effect is desirable, with at least one dose that is above the intended clinical dose. It is preferable that a quantitative PCR assay be used for assessment of vector biodistribution and is validated using PCR as demonstrated in this paper. Towards this end, we assessed the biodistribution of the vector in major organs of the intravitreally injected wild‐type dogs, as well as body wastes and body fluids. Non‐significant levels of the vMCO‐I vector ITRs were observed in some dog tissues during qPCR analysis, but the gels from qPCR and PCR did not show any ITR product (~4 kb). This is probably due to the increased sensitivity of qPCR via use of SYBR Green, or formation of primer‐dimers and higher (forty) cycle numbers.^57^ Nevertheless, qPCR is a sensitive method for demonstrating gene transfer and evaluating biodistribution following AAV‐mediated transduction in vivo.^58^ In this study, we did not find any significant dose‐dependent presence of the vector DNA or transgene in most of the screened tissues. These results suggest that there was limited systemic exposure to vector, or the injected vector was cleared before the time point of analysis (after necropsy).

In summary, intravitreal injection of AAV2‐based vMCO‐I led to cell‐specific expression of MCO in wild‐type dog retina. In addition, no significant inflammation or immune response in retina was observed after vMCO‐I injection into the intravitreal space as demonstrated by immunohistochemistry, suggesting that innate immunity to AAV2 may be insignificant. This result is consistent with a previous study comparing adenoviral vectors and AAV2, which found that the innate immune response to AAV was weak and transient relative to the potent and prolonged response to adenovirus. The absence of significant immune response against intravitreally injected vMCO‐I is important because some human clinical trials and in animal models have been hampered by undesired responses. Although trace amounts of vector DNA were detected in some of the organs, intravitreal injection of vMCO‐I at different doses appears to be safe, with no discernible effects on general health or behaviour of the dogs. Future studies will focus on efficacy of the vMCO‐I in transducing the targeted retinal cells in human and its impact on improving vision in patients with retinal degeneration.

## CONFLICT OF INTEREST

The author Samarendra Mohanty has equity interest in Nanoscope Technologies LLC and Nanoscope Therapeutics Inc. Kissaou Tchedre, Subrata Batabyal, Ananta Ayyagari and Sai Chavala has equity interest in Nanoscope Therapeutics Inc.

## AUTHOR CONTRIBUTIONS

**Kissaou T. Tchedre:** Investigation (lead); Writing‐original draft (equal). **Subrata Batabyal:** Data curation (equal); Investigation (equal); Visualization (equal); Writing‐review & editing (equal). **Melissa Galicia:** Investigation (supporting). **Darryl Narcisse:** Investigation (supporting); Writing‐review & editing (equal). **Sourajit Mitra Mustafi:** Data curation (supporting); Writing‐review & editing (supporting). **Ananta Ayyagari:** Writing‐review & editing (supporting). **Sai Chavala:** Writing‐review & editing (supporting). **Samarendra K. Mohanty:** Conceptualization (lead); Funding acquisition (lead); Supervision (lead); Writing‐review & editing (equal).

## CONSENT FOR PUBLICATION

All authors agreed to the publication.

## Supporting information

Fig S1

Fig S2

Table S1

Table S2

Table S3

Table S4

Table S5

Table S6

Table S7

Table S8

Table S9

Table S10

Table S11

Table S12

Table S13

## Data Availability

Datasets used and/or analysed during the current study are available from the corresponding author on reasonable request.
