# Supplementary material for: Biodistribution of adeno‐associated virus type 2 carrying multi‐characteristic opsin in dogs following intravitreal injection
Source: J Cell Mol Med. 2021 Aug 21;25(18):8676–86. doi: 10.1111/jcmm.16823 (PMC8435460; doi:10.1111/jcmm.16823)
Supplement: Supplementary file 1 — Fig S1 [file JCMM-25-8676-s011.docx]

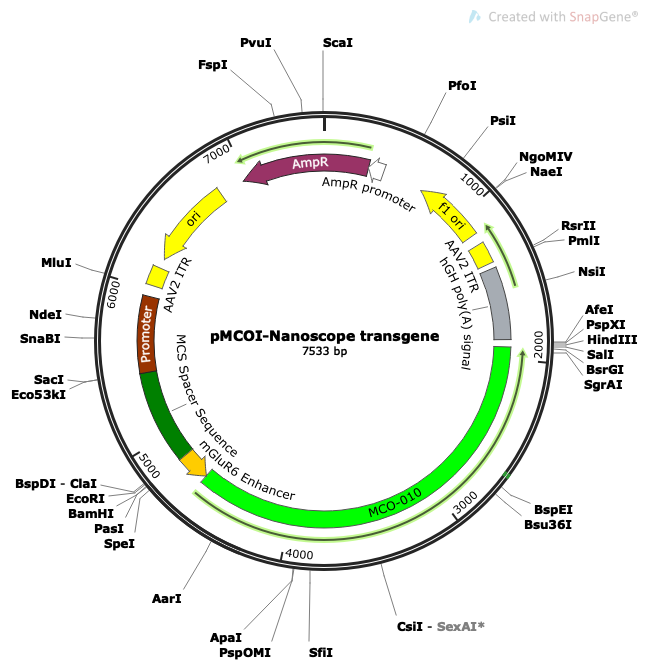


**Supplementary Figure 1. Circular map showing the insertion of MCO-I gene and restriction sites (BamH1 and Sal1)**. MCO-I sequence is published in Non-provisional PCT (PCT/US2017/059922).
