# Supplementary material for: Biodistribution of adeno‐associated virus type 2 carrying multi‐characteristic opsin in dogs following intravitreal injection
Source: J Cell Mol Med. 2021 Aug 21;25(18):8676–86. doi: 10.1111/jcmm.16823 (PMC8435460; doi:10.1111/jcmm.16823)
Supplement: Supplementary file 2 — Fig S2 [file JCMM-25-8676-s007.docx]

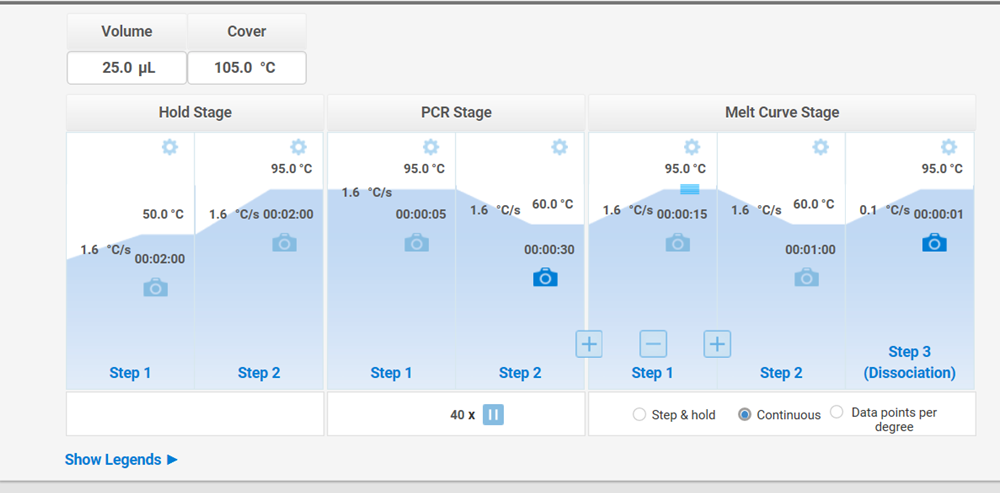


**Supplementary Figure 2. qPCR conditions used for analysis of vMCO-I content in the tissues**. Applied Biosystems QuantStudio 3 qPCR machine was used for this study.
