# Supplementary material for: Biodistribution of adeno‐associated virus type 2 carrying multi‐characteristic opsin in dogs following intravitreal injection
Source: J Cell Mol Med. 2021 Aug 21;25(18):8676–86. doi: 10.1111/jcmm.16823 (PMC8435460; doi:10.1111/jcmm.16823)
Supplement: Supplementary file 3 — Table S1 [file JCMM-25-8676-s015.docx]

| **A: Dose and time of Biodistribution analysis of urine and necropsy samples from vMCO-I injected Dogs using QPCR** | | | |
| --- | --- | --- | --- |
| **Group** | **Dose (75 µL /OD-eye)** | **Time**  **Point** | **List of tissue sample analyzed** |
| 1  (2M + 2F) | 8.6 x 1012 VG/ml AAV- Vehicle | 13 weeks | Lung, Liver, Kidney, Mesenteric and Mandibular Lymph Nodes, Heart, Spleen,  Testis/Ovary, Urine |
| 2  (2M + 2F) | 8.6 x 1012 VG/ml vMCO-I | 13 weeks | Lung, Liver, Kidney, Mesenteric and  Mandibular Lymph Nodes, Heart, Spleen, Testis/Ovary, Urine |
| 3  (2M + 2F) | 1.0 x 1012 VG/ml vMCO-I | 13 weeks | Lung, Liver, Kidney, Mesenteric and Mandibular Lymph Nodes, Heart, Spleen,  Testis/Ovary, Urine |

| **B: Dose and time of Biodistribution of vMCO-I in in-life samples (Feces, Saliva and Nasal Secretions) from Dogs using QPCR** | | | | | |
| --- | --- | --- | --- | --- | --- |
| **Group** | **Dose (75 µL /OD-eye)** | **Time Points** | | | |
| 1  (2M + 2F) | 8.6 x 1012 VG/ml AAV-Vehicle | Baseline | 1 week | 3 weeks | 13 weeks |
| 2  (2M + 2F) | 8.6 x 1012 VG/ml vMCO-I | Baseline | 1 week | 3 weeks | 13 weeks |
| 3  (2M + 2F) | 1.0 x 1012 VG/ml vMCO-I | Baseline | 1 week | 3 weeks | 13 weeks |

**Supplementary Table 1. Dog groups for intravitreal injection and time points for sample collection.** (A) Dose and time of Biodistribution analysis of Samples from vMCO-I injected Dogs using QPCR. (B) Dose and time of Biodistribution of vMCO-I in in-life samples (Feces, Saliva and Nasal Secretions) from Dogs using QPCR. In Group 1, 2 and 3, the OD eyes of dogs were injected intravitreally with 75 µL of Vehicle/vMCO-I and OS eyes were not injected.
