# Supplementary material for: Biodistribution of adeno‐associated virus type 2 carrying multi‐characteristic opsin in dogs following intravitreal injection
Source: J Cell Mol Med. 2021 Aug 21;25(18):8676–86. doi: 10.1111/jcmm.16823 (PMC8435460; doi:10.1111/jcmm.16823)
Supplement: Supplementary file 4 — Table S2 [file JCMM-25-8676-s005.docx]

| **Dog ID** | **Feces Samples** | | | |
| --- | --- | --- | --- | --- |
|  | Baseline | 1 Week | 3 Weeks | 13 Weeks |
| **Group 1: Control AAV2 (8.6x10^12^ VG/ml AAV-vehicle)** | | | | |
| #1001 (Male) | + | + | + | + |
| #1002 (Male) | + | + | + | - |
| #1501 (Female) | + | + | + | + |
| #1502 (Female) | + | + | + | + |
| **Group 2: VMCO-I (8.6x10^12^ VG/ml)** | | | | |
| #2001 (Male) | - | + | + | + |
| #2002 (Male) | - | + | + | + |
| #2501 (Female) | + | + | + | + |
| #2502 (Female) | + | + | + | + |
| **Group 3: VMCO-I (1.0x10^12^ VG/ml)** | | | | |
| #3001 (Male) | + | + | + | + |
| #3002 (Male) | + | + | + | + |
| #3501 (Female) | + | + | + | + |
| #3502 (Female) | + | + | + | + |
| +: vector amplification; -: no amplification | | | | |

**Supplementary Table 2. Longitudinal study of presence of AAV2 packaged Multi-Characteristic Opsin (vMCO-I) in feces**. Detection of AAV vector DNA in dogs’ feces at baseline and three different time points after intravitreal injection. DNA samples were extracted from feces of dogs, wherein the ITR segment of the vector gene was amplified. Though + implies vector amplification in qPCR, the average values are within error range of qPCR assay, which is attributed to sensitivity and variation in sample handling.
