# Supplementary material for: Biodistribution of adeno‐associated virus type 2 carrying multi‐characteristic opsin in dogs following intravitreal injection
Source: J Cell Mol Med. 2021 Aug 21;25(18):8676–86. doi: 10.1111/jcmm.16823 (PMC8435460; doi:10.1111/jcmm.16823)
Supplement: Supplementary file 6 — Table S4 [file JCMM-25-8676-s004.docx]

| **Dog ID** | **Nasal Secretion Samples** | | | |
| --- | --- | --- | --- | --- |
|  | Baseline | 1 Week | 3 Weeks | 13 Weeks |
| **Group 1: Control AAV2 (8.6x10^12^ VG/ml AAV-vehicle)** | | | | |
| #1001 (Male) | - | - | - | - |
| #1002 (Male) | - | - | - | - |
| #1501 (Female) | - | - | - | - |
| #1502 (Female) | - | - | - | - |
| **Group 2: VMCO-I (8.6x10^12^ VG/ml)** | | | | |
| #2001 (Male) | - | - | - | - |
| #2002 (Male) | - | - | - | - |
| #2501 (Female) | - | - | - | - |
| #2502 (Female) | - | - | - | - |
| **Group 3: VMCO-I (1.0x10^12^ VG/ml)** | | | | |
| #3001 (Male) | - | + | - | - |
| #3002 (Male) | - | - | - | - |
| #3501 (Female) | - | - | - | - |
| #3502 (Female) | - | - | - | - |
| +: vector amplification; -: no amplification | | | | |

**Supplementary Table 4. Longitudinal study of presence of AAV2 packaged Multi-Characteristic Opsin (vMCO-I) in nasal secretion**. Detection of AAV vector DNA in dog nasal secretions. DNA samples were extracted from nasal secretion of dogs, wherein the ITR segment of the vector gene was amplified. Though + implies vector amplification in qPCR, the average values are within error range of qPCR assay, which is attributed to sensitivity and variation in sample handling.
