# Supplementary material for: Biodistribution of adeno‐associated virus type 2 carrying multi‐characteristic opsin in dogs following intravitreal injection
Source: J Cell Mol Med. 2021 Aug 21;25(18):8676–86. doi: 10.1111/jcmm.16823 (PMC8435460; doi:10.1111/jcmm.16823)
Supplement: Supplementary file 8 — Table S6 [file JCMM-25-8676-s001.docx]

| **Dog ID** | **Detection of vector DNA in analyzed non-targeted tissue samples** | | | | | | | |
| --- | --- | --- | --- | --- | --- | --- | --- | --- |
|  | Lung | Liver | Kidney | Mesenteric | Mandibular | Heart | Spleen | Testis/Ovary |
| **Group 1: Control AAV2 (8.6x10^12^ VG/ml AAV-vehicle)** | | | | | | | | |
| #1001 (Male) | - | - | - | nd | - | - | + | - |
| #1002 (Male) | - | - | + | nd | nd | - | + | + |
| #1501 (Female) | nd | + | - | - | nd | + | + | + |
| #1502 (Female) | nd | - | nd | nd | + | + | - | nd |
| **Group 2: VMCO-I (8.6x10^12^ VG/ml)** | | | | | | | | |
| #2001 (Male) | nd | + | + | - | + | - | - | + |
| #2002 (Male) | nd | nd | nd | + | nd | + | - | + |
| #2501 (Female) | + | nd | + | + | nd | + | + | + |
| #2502 (Female) | nd | nd | nd | nd | nd | - | - | - |
| **Group 3: VMCO-I (1.0x10^12^ VG/ml)** | | | | | | | | |
| #3001 (Male) | nd | nd | - | - | + | - | + | + |
| #3002 (Male) | nd | nd | nd | nd | - | + | + | - |
| #3501 (Female) | nd | nd | - | - | + | - | + | + |
| #3502 (Female) | nd | - | nd | nd | nd | + | + | + |
| nd: Not Determined; +: vector amplification; -: no amplification | | | | | | | | |

**Supplementary Table 6. Biodistribution of AAV2 packaged Multi-Characteristic Opsin in non-targeted tissues.** qPCR quantification of vector sequences in dogs of group 1, group 2, group 3. Detection of AAV vector DNA in dog tissues, wherein the ITR segment of the vector gene was amplified. Though + implies vector amplification in qPCR, the average values are within error range of qPCR assay, which is attributed to sensitivity and variation in sample handling.
