# Supplementary material for: Biodistribution of adeno‐associated virus type 2 carrying multi‐characteristic opsin in dogs following intravitreal injection
Source: J Cell Mol Med. 2021 Aug 21;25(18):8676–86. doi: 10.1111/jcmm.16823 (PMC8435460; doi:10.1111/jcmm.16823)
Supplement: Supplementary file 9 — Table S7 [file JCMM-25-8676-s008.docx]

| **Primary antibodies** | | | |
| --- | --- | --- | --- |
| Name | Type | Catalog Number | Source |
| mCherry | IgG2a (Host-mouse) | NBP1-96752 | Novus Biological |
| pKCα | IgG (Host-Rabbit) | P45-17551 | Fisher Scientific |
| IFN- γ | IgG (Host-Rabbit) | MBS2027150 | Mybiosource |
| CD45 | IgG (Host: Mouse) | MA5-16603 | Fisher Scientific |

**Supplementary Table 7: List of Primary antibodies Used in this Study**
