# Supplementary material for: Biodistribution of adeno‐associated virus type 2 carrying multi‐characteristic opsin in dogs following intravitreal injection
Source: J Cell Mol Med. 2021 Aug 21;25(18):8676–86. doi: 10.1111/jcmm.16823 (PMC8435460; doi:10.1111/jcmm.16823)
Supplement: Supplementary file 10 — Table S8 [file JCMM-25-8676-s006.docx]

| **Secondary antibodies** | | | |
| --- | --- | --- | --- |
| Name | Type | Catalog Number | Source |
| Dylight 488 | Goat Anti-Rabbit IgG | 35552 | Fisher Scientific |
| Alexa Fluor 488 | Goat Anti-Mouse IgG | A11001 | Fisher Scientific |
| Alexa Fluor 568 | Goat anti-Mouse IgG | A11004 | Fisher Scientific |

**Supplementary Table 8: List of Secondary antibodies used in this Study.**
