# Supplementary material for: Biodistribution of adeno‐associated virus type 2 carrying multi‐characteristic opsin in dogs following intravitreal injection
Source: J Cell Mol Med. 2021 Aug 21;25(18):8676–86. doi: 10.1111/jcmm.16823 (PMC8435460; doi:10.1111/jcmm.16823)
Supplement: Supplementary file 11 — Table S9 [file JCMM-25-8676-s003.docx]

| **Dilution Ratio for Antibodies used (based on literature/manufacturers recommendation)** | | | |
| --- | --- | --- | --- |
| Primary Antibodies | Dilution | Secondary Antibodies | Dilution |
| mCherry | 1: 250 | DyLight 488 | 1: 250 |
| PKCα | 1: 500 | Alexa Fluor 488 | 1: 250 |
| CD45 | 1: 250 | Alexa Fluor 568 | 1: 250 |
| IFN- γ | 1: 250 |  |  |

**Supplementary Table 9: Primary and secondary antibodies’ Dilution Ratio**
