# Supplementary material for: Biodistribution of adeno‐associated virus type 2 carrying multi‐characteristic opsin in dogs following intravitreal injection
Source: J Cell Mol Med. 2021 Aug 21;25(18):8676–86. doi: 10.1111/jcmm.16823 (PMC8435460; doi:10.1111/jcmm.16823)
Supplement: Supplementary file 13 — Table S11 [file JCMM-25-8676-s009.docx]

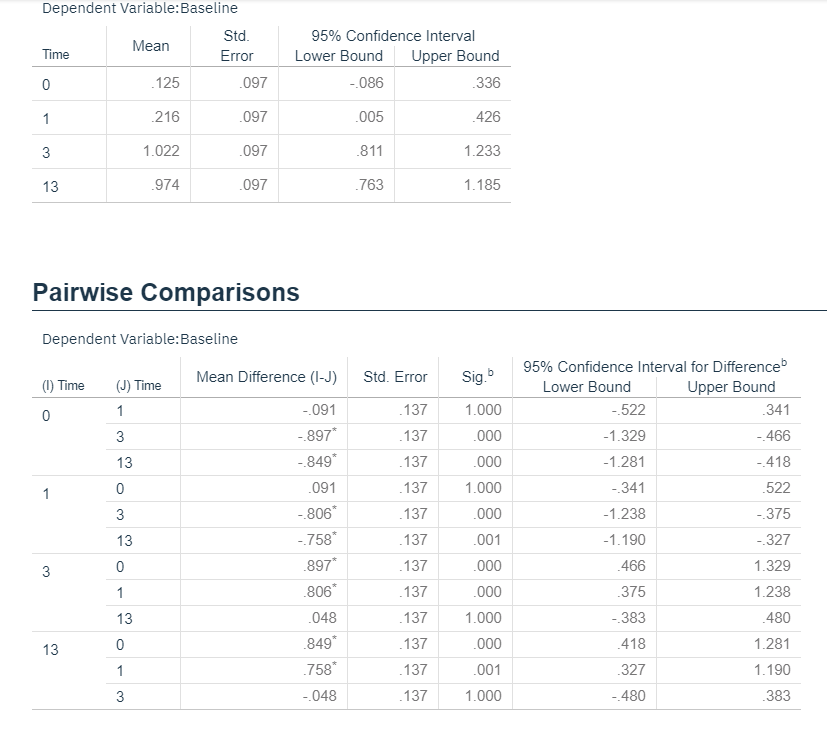


**Supplementary Table 11: SPSS based analysis of differences in presence of vector DNA in feces at different time points within Group-2 using Generalized linear model.** Value=M1*Timeline +constant. The encircled significance values show progressive increase in Vector copy/ng of DNA between 0 (baseline) and after injection time points at 1^st^, 3^rd^ and 13^th^ weeks.
