# Supplementary material for: Biodistribution of adeno‐associated virus type 2 carrying multi‐characteristic opsin in dogs following intravitreal injection
Source: J Cell Mol Med. 2021 Aug 21;25(18):8676–86. doi: 10.1111/jcmm.16823 (PMC8435460; doi:10.1111/jcmm.16823)
Supplement: Supplementary file 15 — Table S13 [file JCMM-25-8676-s014.docx]

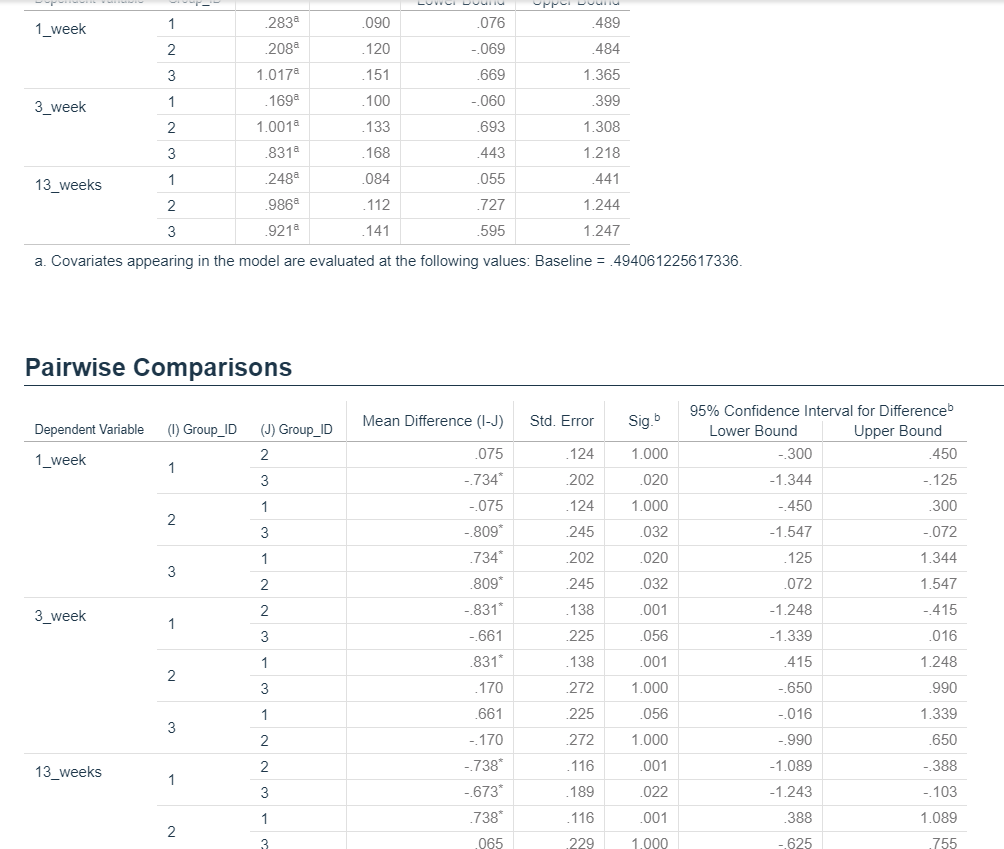


**Supplementary Table 13: SPSS based analysis of group differences at different time points based on Generalized linear model with Baseline as covariate.** Value=M1*Group + M2*Baseline +constant. The encircled significance values show higher values of Vector copy/ng of DNA in Feces at 13^th^ week after injection in the Groups 2 and 3 as compared to group 1.
